# Koch’s Remedy in Relation Specially to Throat Consumption

**Published:** 1891-06

**Authors:** 


KOCH'S REMEDY IN RELATION SPECIALLY TO THROAT CONSUMPTION. By LENNOX BROWNE, F. R. C. S., Ed., Senior Surgeon to the Central London Throat, Nose, and Ear Hospital; author of "The Throat and Nose, and their Diseases," etc. Illustrated by thirty-one cases and by fifty original engravings and diagrams. Philadelphia: Lea Brothers & Co. 1891.

This is a valuable contribution to the study of the important question, What is the proper application of Koch's lymph in the treatment of tuberculosis? It takes, on the whole, a favorable view, and, in this respect, coincides with the opinion of Sir Morell Mackenzie, who, in his last paper, says:

> Whilst the injection of Koch's fluid may not have deserved the enthusiastic reception it first met with, it does not merit its present obloquy. Professor Koch has made a most important discovery; and, when the details as regards dosage have been thoroughly worked out, and conclusions have been arrived at as to the best kind of remedies, constitutional and local, to combine with it, tuberculin will prove a most valuable addition to the curative agencies in the hands of physicians.

Mr. Browne believes from his own experience, that phthisis, in the beginning, can be cured with certainty with this remedy; and this statement is made in large type, so as to impress it upon the mind of the reader. These conclusions from, perhaps, the two best-known laryngologists in England, should, and no doubt will, receive careful attention. At the same time, so many warning voices have been raised concerning this treatment, that our opinion should take, at the present time, the form of the Scotch verdict, "Not proven." Mr. Browne gives his experience in a very pleasing and thorough way. The cases are well presented, and illustrated with charts and drawings, showing the laryngeal appearances from time to time. This we believe to be the most valuable part of the book, and the part to be most consulted. Next to the report of cases, the chapter on Indications and Counter-indications will receive the most attention. The lymph should not be used in the very young or the very old; indeed, Browne thinks it doubtful whether it should be administered much after middle life, on account of its potent influence on the higher nerve centers. Alcoholism, plumbism, and other constitutional diseases engendered by habit of life and by occupation, render its employment doubtful.

Syphilis, on the contrary, is not a counter-indication. Besides these, many questions occur in connection with the tuberculous process itself — its location, its extent, etc. — that materially modify the selection of the lymph treatment. So far as location is concerned, Browne believes that in throat consumption the remedy is especially useful. Upon reading this work, we are more than ever impressed that this whole matter is still in the experimental stage. The contribution of Mr. Browne is, however, of great merit. It is such work that will finally place us in a position to know just the application to make of Koch's lymph in the treatment of consumption.

